# Peripheral Intravenous Catheter Care at Australian Emergency Departments: A Cross‐Sectional Observational Study

**DOI:** 10.1111/jan.16810

**Published:** 2025-02-10

**Authors:** Hui (Grace) Xu, Anna Doubrovsky, Claire M. Rickard, Lauryn Rockliff, Christopher Tang, Amanda J. Ullman

**Affiliations:** ^1^ School of Nursing Queensland University of Technology Brisbane Queensland Australia; ^2^ Nursing & Midwifery Research Centre Royal Brisbane and Women's Hospital Brisbane Queensland Australia; ^3^ Emergency Department Queen Elizabeth II Jubilee Hospital Brisbane Queensland Australia; ^4^ Alliance for Vascular Access Teaching and Research, Schools of Nursing and Midwifery and Pharmacy and Medical Sciences Griffith University Brisbane Australia; ^5^ School of Nursing, Midwifery and Social Work The University of Queensland Brisbane Queensland Australia; ^6^ Herston Infectious Diseases Institute Metro North Health Brisbane Australia; ^7^ NHMRC Centre of Research Excellence (CRE) in Wiser Wound Care, Menzies Health Institute Queensland Griffith University Brisbane Queensland Australia; ^8^ Department of Emergency Department Logan Hospital Brisbane Australia; ^9^ Department of Emergency Department Redland Hospital Brisbane Australia; ^10^ Children's Health Queensland Hospital and Health Service Brisbane Queensland Australia

**Keywords:** clinical care standard, emergency care, guideline adherence, observation study, peripheral intravenous catheters

## Abstract

**Background:**

Peripheral intravenous catheters (PIVCs) serve as crucial devices for essential care administration in emergency departments (ED). In Australia, to standardise clinical practice, the national PIVC Clinical Care Standard was introduced in 2021, however adherence to the Standard has not been adequately explored. Therefore, this study aims to investigate ED clinicians' adherence to the Standard via prospective audit.

**Method:**

This cross‐sectional observational study of PIVCs was conducted in three Australian EDs between 2022 and 2023. Data were collected in alignment with the quality indicators in the PIVC Clinical Care Standard. Research nurses collected the data from bedside observation and chart audit, with data analysed descriptively.

**Findings:**

Out of 1568 episodes of PIVC care recorded, there were notable shortcomings. ED nurses and doctors provided minimal patient partnership during insertion episodes: PIVC self‐care education (*n* = 4, 1.4%), discussion of potential risks/benefits (*n* = 8, 2.9%), and reporting of concerns (*n* = 16, 5.8%). Insertions primarily occurred at the antecubital fossa (*n* = 225, 81.2%), with a common issue being inadequate time for antiseptic solution to air dry (*n* = 156, 56.3%). Ongoing needs assessment was unable to be assessed due to documentation limitations, which were generally incomplete. Idle catheters (inserted but not used) were prevalent (*n* = 115, 41.8%), and only a quarter of inpatient ward admissions (*n* = 75, 27.3%) had clear indications for PIVC use.

**Conclusion:**

These findings highlight the suboptimal ED PIVC practices that require attention and improvement. Innovative interventions and technology are necessary to address some of these suboptimal practices due to their complexity and persistent challenges, despite previous efforts by clinicians and researchers.

**Implications for the Profession and Patient Care:**

The findings underscore the need for well‐resourced efforts to ensure adherence to evidence‐based practices in dynamic clinical settings.

**Reporting Method:**

The study is reported following the Strengthening the Reporting of Observational Studies in Epidemiology (STROBE) Statement.

**Patient or Public Contribution:**

None.

## 1 Introduction

Peripheral intravenous catheters (PIVCs) are widely used in emergency departments (EDs), with 60% of patients receiving at least one PIVC (Hawkins et al. 2018; Alexandrou et al. 2015). As a vascular access device, PIVCs offer reliable access for administering intravenous medications, fluids and diagnostic tests crucial for patient care. Despite their common use, PIVCs are not without complications. A recent systematic review demonstrated that 36.4% of PIVCs failed before treatment completion (Marsh et al. 2024), which impacts patient care and increases organisational costs. Hence, it is imperative for healthcare workers to adhere to recommended evidence and guidelines when managing PIVCs.

Over the past decade, it has become increasingly recognised that poor guideline adherence is a common and serious issue in healthcare, with adherence rates varying widely (10%–80%) depending on the context and evidence (Arts et al. 2016). Therefore, accurately measuring the prevalence of suboptimal guideline adherence is crucial for identifying issues in daily practice and developing strategies to address this common issue. A landmark study estimates that 30% of healthcare provided to patients is of low value or wasteful, and 10% is harmful (Braithwaite et al. 2020), emphasising the importance of implementing evidence‐based practices for meaningful and useful patient care, especially given the current challenging global economic environment.

Multiple international studies have shown that clinicians globally often do not follow recommended practices in both adult and paediatric settings regarding PIVC care, indicating the prevalence of suboptimal PIVC practices (Alexandrou et al. 2018; Ullman et al. 2020), which extends to ED settings as well (Xu et al. 2023). Low compliance with guideline‐recommended practices likely leads to preventable complications, suboptimal patient outcomes and resource wastage (Tuffaha et al. 2014), highlighting the need for knowledge translation to bridge the evidence‐to‐practice gap and improve patient outcomes while saving organisational costs.

### 1.1 Background

Approximately one out of every three PIVCs inserted in the hospital environment is inserted by ED clinicians. These frontline healthcare workers are often regarded as expert inserters for this fundamental clinical task. However, despite its frequency, two out of every three insertion attempts fail, with some patients enduring more than 10 attempts to secure a PIVC (Reigart et al. 2012). Studies have reported that 30%–50% of hospital patients are identified with difficult venous access (DIVA) (Rippey et al. 2016; Whalen et al. 2017). This leads to patient distress and dissatisfaction. An international patient survey demonstrated that over 50% of patients described the PIVC insertion experience as causing moderate pain and leading to a negative hospital experience (Cooke et al. 2018). Despite the stress caused by insertion, half of the PIVCs inserted in ED remain unused (Limm et al. 2013), highlighting the need for regulating ED clinicians' practices.

Addressing these suboptimal practices was the primary motivation behind the development of the first international and national PIVC Clinical Standard. Released by the Australian Commission on Safety and Quality in Health Care in May 2021, the Standard aims to guide clinicians' PIVC practices. However, despite being a mandatory clinical care standard, a recent national survey revealed that 65% of 433 ED nurses and doctors in Australia were unaware of its existence and recommendations (Xu et al. 2023). Their self‐reported practices in the survey, including idle catheter insertion, use of the cubital fossa, and poor documentation, highlighted the suboptimal PIVC practices that are commonly and widely accepted in Australian EDs (Xu et al. 2023; Xu et al. 2023). However, the survey might not fully reflect the true state of these issues due to the tendency of self‐reporting to overestimate compliance and response bias (Wang et al. 2004). Moreover, many previous studies have relied on retrospective chart reviews, which may not accurately capture real practices and hinder the identification of practice gaps due to incomplete and inaccurate documentation by clinicians (Thomas et al. 2020). In response, we designed a prospective, cross‐sectional observational design to reveal real‐world PIVC practices in ED settings.

## 2 The Study

### 2.1 Aim

Our study aims to investigate ED clinicians' adherence to the Standard via prospective audit in multiple Australian EDs.

## 3 Methods

### 3.1 Study Design, Sites and Participants

This prospective, cross‐sectional observational study was conducted at two metropolitan hospitals and one regional hospital in Queensland, Australia. These EDs care for both adult and paediatric patients, with annual ED admissions ranging from 59,865 to 106,415 presentations between 2022 and 2023 (AIHW 2023). Ethics approval was obtained from the Griffith University Human Ethics Committee (2022/025) and Metro South Ethics Committee (HREC/2022/QMS/81524). The findings were reported following the Strengthening the Reporting of Observational Studies in Epidemiology (STROBE) Statement (Elm et al. 2014). The participants included all ED nurses and doctors who inserted PIVCs and provided written consent to allow their practice to be observed by a research nurse (ReN). Causal staff were excluded. The study was advertised via emails, posters and face‐to‐face promotion sessions during staff handover time.

### 3.2 Data Collection and Rigour

The research teams, comprised of ED or PIVC clinical and research experts, developed the data collection form. The contents of the form were designed based on nine of the ten quality indicators outlined in the PIVC Clinical Care Standard (Australian Commission on Safety and Quality in Health Care 2021). We did not use Standard 9 (review of ongoing needs) in this audit. Prior to use, three external clinical and/or research experts reviewed the form for face validity and established its readability and feasibility (Connell et al. 2018). Several minor changes were made to the data collection form based on their recommendations.

Data collection comprised three components: Bedside observation of insertions, bedside observation of PIVC maintenance care, and documentation audits via an electronic medical record system (iEMR). Upon obtaining clinicians' consent for each insertion episode, ReNs collected the clinician's professional characteristics (e.g., profession, clinical role, years of PIVC insertion experience, self‐ranked level of PIVC insertion competency) in a private setting before entering patients' cubicles. Subsequently, ReNs observed clinicians' PIVC insertion practices at the bedside and recorded the data using the data collection form integrated into REDCap (V14.0.15) (Harris et al. 2019), an electronic clinical data management system. For maintenance episodes, ReNs inspected patients who had been in the departments for several hours to physically assess their PIVCs for dressing and complications. ReNs conducted audits of PIVC insertion, maintenance and removal care documentation by retrieving data from iEMR.

The researcher (GX) provided training to ReNs to ensure protocol compliance, thereby ensuring assessor validity and inter‐rater reliability. Any discrepancies noted during the initial audit training were addressed through discussion until a consensus was reached, ensuring assessment consistency and accuracy. Additionally, the researcher (GX) monitored the quality of study data throughout the data collection.

Between October 2022 and May 2023, ReNs were rostered Monday to Friday for morning and afternoon shifts. All clinicians (registered nurses and doctors) who provided informed consent for auditing were monitored during their insertion procedures and subsequent phases of care.

### 3.3 Statistical Analysis

Data were downloaded from RedCap (V14.0.15) (Harris et al. 2019) and imported into RStudio (2022.07.1) (Rstudio: RStudio Team 2019) for analysis with R (V4.2.1) (R: R Core Team 2020). Data were reported descriptively, as per data characteristics. This included participant demographics and responses using percentages for categorical data and mean and standard deviation (SD) or median and interquartile range (IQR) for continuous variables depending on the distribution. No statistical inference or sample size calculations were conducted, as comparisons between groups were not performed. The smallest observed group (*n* = 275, out of 15,000 ED presentations during the study time period) (AIHW 2023) had a margin of error of 5.8%. Consequently, a proportion of 50% for this group corresponds to a 95% confidence interval of 46.49%–53.51% (Qualtrics 2025).

## 4 Findings

A total of 1568 episodes of care were documented, comprising 724 bedside observations and 844 chart audits (reviewing clinicians' notes via the Integrated electronic medical record (ieMR)) across three hospitals (Hospital A = 428 episodes, Hospital B = 755 episodes, and Hospital C = 323 episodes). These episodes encompassed various aspects, including insertion care (552 episodes), maintenance care (741 episodes), and PIVC removal care (275 episodes). Most observations were conducted in acute cubicles (*n* = 106, 38.3%) and fast track (*n* = 58, 20.9%). Emergency insertions accounted for 2.9% (*n* = 8) of the total insertions. Most observed insertions (*n* = 217, 78.3%) were performed during morning shifts.

Table 1 summarises the demographics of inserters. More than half of the observations (*n* = 162, 58.5%) were conducted on nurses. Registered nurses (*n* = 146, 52.7%) and senior medical staff (*n* = 62, 22.4%) were the primary inserters. On average, inserters had 6.3 years (SD = 5.6) of PIVC insertion experience. The findings of observations and chart audits, aligning with the 10 standards, are detailed in Tables 2, 3, 4.

**TABLE 1:** Inserter Demographics.

PIVC Inserters (observations) (*n* = 277)		Hospital A (*N* = 70)	Hospital B (*N* = 112)	Hospital C (*N* = 95)	Total
Clinical Role					
Nurse		49 (70.0%)	72 (64.3%)	41 (43.2%)	162 (58.5%)
Doctor		21 (30.0%)	40 (35.7%)	54 (56.8%)	115 (41.5%)
Clinical Position					
Nursing	Registered Nurse	46 (65.7%)	65 (58.0%)	35 (36.8%)	146 (52.7%)
	Clinical Nurse	2 (2.9%)	3 (2.7%)	4 (4.2%)	9 (3.2%)
	Clinical Nurse Consultant/Educator/ Nurse Practitioner	0 (0%)	2 (1.8%)	2 (2.1%)	4 (1.4%)
Medical	Intern	3 (4.3%)	2 (1.8%)	9 (9.5%)	14 (5.1%)
	Junior House Officer	2 (2.9%)	12 (10.7%)	2 (2.1%)	16 (5.8%)
	Senior House Officer	2 (2.9%)	4 (3.6%)	20 (21.1%)	26 (9.4%)
	Senior medical staff	15 (21.4%)	24 (21.5%)	23 (24.2%)	62 (22.4%)
Years of PIVC Insertion Experience, Mean (SD)		5.1 (3.7)	7.0 (6.6)	6.2 (5.2)	6.3 (5.6)

Abbreviations: PIVC: peripheral intravenous catheter; SD: standard deviation.

**TABLE 2:** Quality Statements 1–4.

Insertion	Hospital A (*N* = 70)	Hospital B (*N* = 112)	Hospital C (*N* = 95)	Total (*N* = 277)
Quality statement 1: Assess intravenous access needs (observations) (*n* = 277)				
PIVC Insertion Purpose				
IV fluid	5 (7.1%)	5 (4.5%)	6 (6.3%)	16 (5.8%)
IV medication	29 (41.4%)	47 (42.0%)	8 (8.4%)	84 (30.3%)
IV contrast	1 (1.4%)	5 (4.5%)	2 (2.1%)	8 (2.9%)
Blood test only	13 (18.6%)	25 (22.3%)	72 (75.8%)	110 (39.7%)
High risk of deterioration^a^	13 (18.6%)	25 (22.3%)	72 (75.8%)	59 (21.3%)
Quality Statement 2: inform and partner with patients (observations) (*n* = 277)				
Inserter explained the reason for the insertion	59 (84.3%)	99 (88.4%)	95 (100%)	253 (91.3%)
Inserter explained insertion risks and benefits	5 (7.1%)	2 (1.8%)	1 (1.1%)	8 (2.9%)
Consent for insertion was obtained	69 (98.6%)	108 (96.4%)	95 (100%)	272 (98.2%)
Inserter asked patients about their PIVC history	38 (54.3%)	34 (30.4%)	54 (56.8%)	126 (45.5%)
Inserter instructed the patient to report PIVC‐related concerns	0 (0%)	11 (9.8%)	5 (5.3%)	16 (5.8%)
Inserter provided education on PIVC self‐care	1 (1.4%)	2 (1.8%)	1 (1.1%)	4 (1.4%)
Quality statement 3: Ensure competency (observations) (*n* = 277)				
Inserter self‐rated PICV Insertion Competency				
Learner	8 (11.4%)	12 (10.7%)	14 (14.7%)	34 (12.3%)
Competent inserter	31 (44.3%)	47 (42.0%)	16 (16.8%)	94 (33.9%)
Advanced inserter	22 (31.4%)	25 (22.3%)	15 (15.8%)	62 (22.4%)
Expert inserter	9 (12.9%)	28 (25.0%)	50 (52.6%)	87 (31.4%)
Inserter completed PIVC Course and Practice session	65 (92.9%)	100 (89.3%)	89 (93.7%)	254 (91.7%)
Quality statement 4: Choose the right insertion site and PIVC (observations) (*n* = 277)				
PIVC inserted on the dominant arm	43 (61.4%)	53 (47.3%)	52 (54.7%)	148 (53.4%)
Inserter checked for contra‐indications	5 (7.1%)	38 (33.9%)	0 (0%)	43 (15.5%)
Inserted over an area of flexion				
Antecubital fossa	57 (81.4%)	83 (74.1%)	84 (88.4%)	224 (80.9%)
Wrist area	4 (5.7%)	3 (2.7%)	2 (2.1%)	9 (3.2%)

Abbreviations: IV: intravenous; PIVC: peripheral intravenous catheter; SD: standard deviation.

^a^ High risk, potential clinical deterioration, abnormal parameters, unwell patients.

**TABLE 3:** Quality Statements 5–6.

Pivc Insertion	Hospital A (*N* = 70)	Hospital B (*N* = 112)	Hospital C (*N* = 95)	Total
Quality statement 5: Maximise first insertion success (observations) (*n* = 277)				
Inserter identified as DIVA patient	13 (18.6%)	19 (17.0%)	16 (16.8%)	48 (17.3%)
Used ultrasound to assist insertion	5 (7.1%)	10 (8.9%)	3 (3.2%)	18 (6.5%)
Used near‐infrared vessel visualisation device	0 (0%)	0 (0%)	2 (2.1%)	2 (0.7%)
Escalated to a more experienced insertor after two failed attempts	1 (1.4%)	3 (2.7%)	4 (4.2%)	8 (2.9%)
Total number of PIVC insertion attempts, Mean (SD)	1.20 (0.499)	1.35 (0.768)	1.26 (0.530)	1.28 (0.631)
Quality statement 6: Insert and secure (observations) (*n* = 277)				
Gloves used	68 (97.1%)	110 (98.2%)	94 (98.9%)	272 (98.2%)
Goggles Used	3 (4.3%)	28 (25.0%)	12 (12.6%)	43 (15.5%)
Cleaned the trolley with antiseptic wipes	17 (24.3%)	36 (32.1%)	28 (29.5%)	81 (29.2%)
Five moments of hand hygiene				
Moment 1—before touching patient	60 (85.7%)	103 (92.0%)	85 (89.5%)	248 (89.5%)
Moment 2—before a procedure	59 (84.3%)	100 (89.3%)	86 (90.5%)	245 (88.4%)
Moment 3—after a procedure	39 (55.7%)	46 (41.1%)	66 (69.5%)	151 (54.5%)
Moment 4—after touching patient	43 (61.4%)	49 (43.8%)	67 (70.5%)	159 (57.4%)
Moment 5—after touching patient's surroundings	44 (62.9%)	60 (53.6%)	61 (64.2%)	165 (59.6%)
Aseptic technique maintained				208 (75.1%)
Main reasons for not maintaining an aseptic technique				
Placed nonsterile items into a sterile field	7 (10.0%)	5 (4.46%)	0 (0.0%)	12 (4.3%)
Touched insertion site after cleaning with alcohol wipes	22 (31.4%)	13 (11.6%)	19 (20.0%)	54 (19.5%)
Other	0 (0.0%)	3 (2.7%)	0 (0.0%)	3 (1.1%)
PIVC pack used	65 (92.9%)	103 (92.0%)	94 (98.9%)	262 (94.6%)
Used 2% chlorhexidine + 70% alcohol to clean insertion site	70 (100%)	111 (99.1%)	95 (100%)	276 (99.6%)
Allowed the alcohol to become completely air dry before accessing	60 (85.7%)	77 (68.8%)	19 (20.0%)	156 (56.3%)
Mean waiting time (SD) (seconds)				7.57 (1.89)
PIVC size				
18 gauge	3 (4.3%)	7 (6.3%)	10 (10.5%)	20 (7.2%)
20 gauge	60 (85.7%)	94 (83.9%)	80 (84.2%)	234 (84.5%)
22 gauge	7 (10.0%)	11 (9.8%)	5 (5.3%)	23 (8.3%)
Insertion site				
ACF	58 (82.9%)	83 (74.1%)	84 (88.4%)	225 (81.2%)
Forearm	3 (4.3%)	8 (7.1%)	4 (4.2%)	15 (5.4%)
Wrist	4 (5.7%)	4 (3.6%)	2 (2.1%)	10 (3.6%)
Hand	4 (5.7%)	13 (11.6%)	5 (5.3%)	22 (7.9%)
Upper arm	1 (1.4%)	3 (2.7%)	0 (0%)	4 (1.4%)
IV extension line was clamped	33 (47.1%)	56 (50.0%)	54 (56.8%)	143 (51.6%)
Safe disposal of sharps	63 (90.0%)	108 (96.4%)	95 (100%)	266 (96.0%)
Label and date the PIVC site	14 (20.0%)	40 (35.7%)	43 (45.3%)	97 (35.0%)

Abbreviations: ACF: antecubital fossa; IV: intravenous; PIVC: peripheral intravenous catheter; SD: standard deviation.

**TABLE 4:** Quality Statements 7, 8 and 10.

Maintenance and removal	Hospital A	Hospital B	Hospital C	Total
Standard 7: Document decisions and care (chart audits) (*n* = 275)				
Numbers per hospital	*N* = 131	*N* = 133	*N* = 11	*N* = 275
PIVC insertion indication recorded	76 (58.0%)	89 (66.9%)	9 (81.8%)	174 (63.3%)
PIVC gauge recorded	72 (55.0%)	77 (57.9%)	2 (18.2%)	151 (54.9%)
Insertion site recorded	80 (61.1%)	79 (59.4%)	8 (72.7%)	167 (60.7%)
Inserter recorded	50 (38.2%)	56 (42.1%)	7 (63.6%)	113 (41.1%)
Number of insertion attempts recorded	47 (35.9%)	63 (47.4%)	4 (36.4%)	114 (41.5%)
Date and time of insertion recorded	70 (53.4%)	80 (60.2%)	7 (63.6%)	157 (57.1%)
PIVC‐related complications recorded	48 (36.6%)	33 (24.8%)	1 (9.1%)	82 (29.8%)
PIVC removal recorded	2 (1.5%)	3 (2.3%)	4 (36.4%)	9 (3.3%)

Maintenance and removal	Hospital A	Hospital B	Hospital C	Total
Standard 8: Routine use: inspect, access and flush(observations) (*n* = 447)				
Numbers per hospital	*N* = 70	*N* = 209	*N* = 106	*N* = 447
PIVC Dressing Inspection				
Dry	68 (97.1%)	204 (98.6%)	162 (96.4%)	434 (97.5%)
Intact	58 (82.9%)	195 (94.2%)	164 (97.6%)	417 (93.7%)
Reinforcement applied	8 (11.4%)	35 (16.9%)	23 (13.7%)	66 (14.8%)
Signs of complications				
Phlebitis	3 (4.3%)	1 (0.5%)	4 (2.4%)	8 (1.8%)
Extravasation	0 (0%)	0 (0%)	1 (0.6%)	1 (0.2%)

Maintenance and removal	Hospital A	Hospital B	Hospital C	Total
Standard 8: Routine use: inspect, access and flush (chart audits) (*n* = 294)				
Numbers per hospital	*N* = 67	*N* = 202	*N* = 25	*N* = 294
PIVC Site inspection recorded on each shift	35 (52.2%)	129 (63.9%)	11 (44.0%)	175 (59.5%)
PIVC Condition recorded on each shift	37 (55.2%)	115 (56.9%)	11 (44.0%)	163 (55.4%)
PIVC Site and Patency check recorded on each shift	36 (53.7%)	117 (57.9%)	10 (40.0%)	163 (55.4%)

Maintenance and removal	Hospital A	Hospital B	Hospital C	Total
Standard 10: Remove safely and replace if needed (chart audits) (*n* = 275)				
Numbers per hospital	*N* = 90	*N* = 99	*N* = 86	*N* = 275
PIVC was used in ED	64 (71.1%)	62 (62.6%)	34 (39.5%)	160 (58.2%)
PIVC likely to be used in inpatient ward with prescribed IV antibiotics and IV fluid	37 (41.1%)	25 (25.3%)	13 (15.1%)	75 (27.3%)

Abbreviations: IV: intravenous; PIVC: peripheral intravenous catheter; SD: standard deviation.

### 4.1 Standard 1—Assess Intravenous Access Needs

Most PIVCs were inserted for blood tests (*n* = 110, 39.7%), intravenous medications (*n* = 84, 30.3%) or for high risk of potential clinical deterioration (*n* = 59, 21.3%).

### 4.2 Standard 2—Inform and Partner With Patients

While 91.3% of inserters (*n* = 253) explained the reason for insertion and 98.2% (*n* = 272) obtained patient consent, only 2.9% (*n* = 8) discussed the potential risks and benefits associated with PIVC insertions. Less than half (*n* = 126, 45.5%) asked about the patients' PIVC history, and very few instructed patients to report PIVC‐related concerns (*n* = 16, 5.8%) or provided PIVC self‐care education (*n* = 4, 1.4%).

### 4.3 Standard 3—Ensure Competency

Most inserters rated themselves as competent (*n* = 94, 33.9%) or expert inserters (*n* = 87, 31.4%). Almost all clinicians (*n* = 254, 91.97%) had completed Queensland Health's credentialed PIVC courses and practice sessions.

### 4.4 Standard 4—Choose the Right Insertion Site and Device

Most PIVCs were placed over an area of flexion, with 81.2% (*n* = 225) in the antecubital fossa and over half (*n* = 148, 53.4%) in the dominant arm. Only 15.5% (*n* = 43) of inserters checked for a contraindication to particular sites.

### 4.5 Standard 5—Maximise First Insertion Success

Although 17.3% of insertions (*n* = 48) were identified as DIVA, only 6.5% (*n* = 18) had ultrasound used and 0.7% (*n* = 2) used near‐infrared visualisation devices. Despite known likely insertion challenges, only 2.9% (*n* = 8) were escalated to more experienced inserters.

### 4.6 Standard 6—Insert and Secure

Almost all inserters (*n* = 272, 98.2%) wore gloves. Less than a third (*n* = 81, 29.2%) cleaned the PIVC trolley with antibacterial wipes before or after insertions. Regarding hand hygiene, most inserters followed protocols before touching patients (*n* = 248, 89.5%) or before a procedure (*n* = 245, 88.4%), but only half did so after the procedure (*n* = 151, 54.5%), after touching patients (*n* = 159, 57.4%) and their surroundings (*n* = 165, 59.6%). Three‐quarters (*n* = 208, 75.1%) maintained an aseptic technique during insertions. The primary reasons for not maintaining an aseptic technique were touching the insertion site after cleaning with alcohol wipes (*n* = 54, 19.5%) and placing nonsterile items into a sterile field (*n* = 12, 4.3%). Almost all insertions (*n* = 276, 99.6%) used 2% chlorhexidine and 70% alcohol to clean the insertion site, though only half of them (*n* = 156, 56.3%) waited (7.57 s, SD = 1.89) for the antiseptic solution to air dry completely before insertion. Size 20 gauge (*n* = 234, 84.5%) was the most used PIVC. The antecubital fossa (ACF) (*n* = 225, 81.2%) was the most used insertion site, followed by the hand (*n* = 22, 7.9%) and forearm (*n* = 15, 5.4%). Only a third (*n* = 97, 35.0%) of insertions were labelled and dated.

### 4.7 Standard 7—Document Decisions and Care

Based on 275 chart audits, two‐thirds (*n* = 174, 63.3%) recorded insertion indications. More than half documented the PIVC gauge (*n* = 151, 54.9%), insertion site (*n* = 167, 60.7%), and date and time of insertion (*n* = 157, 57.1%). However, fewer than half recorded inserter details (*n* = 113, 41.1%) or number of insertion attempts (*n* = 114, 41.5%). Less than a third (*n* = 82, 29.8%) documented PIVC‐related complications and only 3.3% (*n* = 9) recorded time of removal.

### 4.8 Standard 8—Routine Use: Inspect, Access and Flush

Among 447 episodes of maintenance care observed, most PIVC dressings were dry (97.5%) and intact (*n* = 417, 93.7%), with 14.8% reinforced with tape or bandages. Few showed signs of phlebitis (*n* = 8, 1.8%) or extravasation (*n* = 1, 0.2%). From chart audits of 294 charts, more than half recorded PIVC site inspection (*n* = 175, 59.5%), condition (*n* = 163, 55.4%), and site and patency check (*n* = 163, 55.4%) during each shift.

### 4.9 Standard 10—Remove Safely and Replace if Needed

Based on 275 chart audits, just over half (*n* = 160, 58.2%) of the PIVCs were used and nearly half PIVCs (*n* = 115, 41.8%) were idle catheters. Among those patients who were admitted into inpatient wards, only 27.3% (*n* = 75) were likely to be used in patient wards with prescribed intravenous medications and fluid.

## 5 Discussion

This cross‐sectional observational study conducted in three Australian EDs analysed 1568 care episodes, covering the entire PIVC care process from insertion to removal. It offers new insights, as previous studies in EDs primarily focused on insertion alone. In this study, we observed 277 insertion episodes, with a balanced distribution between nurses and doctors (58.5% vs. 41.5%), providing a comprehensive view of insertion practices. Key areas for improvement include patient partnership, insertion practice, and documentation. While some areas, like insertion site disinfection, could benefit from simple interventions like education and training, others, such as idle catheter (suggesting ‘just in case’ insertions), ACF insertion, and inadequate patient partnership, require innovative interventions and technology due to their complexity and persistent challenges despite previous efforts by clinicians and researchers (Hawkins et al. 2018; Lim et al. 2020; Clinical Excellence Queensland 2020).

Previous research has not explored the role of informing and partnering with patients in PIVC care, especially in the ED setting. Our study makes a significant contribution to this gap, as patient engagement during PIVC insertion is a key care quality indicator in the national PIVC Clinical Care Standard (Indicator 2) (Australian Commission on Safety and Quality in Health Care, 2022). We identified notable deficiencies: Only 2.9% of ED clinicians discussed potential risks and benefits, 5.8% prompted patients to report concerns, and merely 1.4% provided PIVC self‐care education. Patient engagement is crucial to ensuring safety and meeting quality health service standards both internationally and nationally (Australian Commission on Safety and Quality in Health Care 2021; Care Quality Commission 2014; Agency for Healthcare Research and Quality 2013). Patients' perspectives on PIVC care should not be overlooked, highlighting the necessity of active patient participation for achieving shared clinical decision‐making. Previous studies have shown that patient engagement has a positive impact on service redesign, care delivery, and patient experience, leading to empowered patients and optimising care outcomes (Bombard et al. 2018; Marzban et al. 2022). Given the central role of patients in care, future studies should prioritise patient involvement and develop effective strategies for their participation in PIVC care.

Idle catheter insertion remains a significant concern during our study's insertion phase. We found that 41.8% of PIVCs were not utilised, which is consistent with findings from prior ED studies indicating that nearly half of PIVCs were inserted but not used (Limm et al. 2013). Previous research has highlighted instances where PIVCs were inserted but not used due to patient condition instability and the risk of deterioration (Thomas et al. 2020). In our study, only 21.3% of PIVCs were inserted for these reasons. Despite the promotion of the ‘80% sure rule’ (Kelly 2014) in Australia, particularly through initiatives like the CREDIT project by the Promoting Value‐based Care in EDs (PROV‐ED) statewide champion (Clinical Excellence Queensland 2020), a high percentage (39.7%) of PIVCs were still inserted for blood tests only in our study. Similarly, projects carried out in Cananda and the Netherlands aimed at reducing idle catheter use showed some effects (Rangarajan et al. 2018; Laan et al. 2020). Although these champions and researchers may have influenced clinician practice temporarily, the change was not sustained during our study period. This highlights the need for more effective and enduring interventions or decision‐making tools to address this issue. The complexity of the ‘80% sure rule’ (Kelly 2014) with multiple criteria may pose challenges, especially for junior clinicians, underscoring the necessity for user‐friendly decision‐making tools in future studies to improve insertion decision‐making and mitigate suboptimal idle catheter insertion practices.

Our study has identified further concerns regarding ACF insertion. A comparison with a recent national survey (Xu et al. 2023) involving over 440 ED clinicians revealed a significant difference in ACF insertion rates (81.2% audits vs. 41% survey). This pattern is consistent with the findings from a systematic review (Mickan et al. 2011), which suggested that adherence to guideline recommendations tends to be lower in practice than what is reported in self‐report data. Clinicians often overestimate their adherence and compliance due to bias (Mickan et al. 2011; Blonde et al. 2018). ACF insertion (81.2%) is a persistent issue in ED practice, as highlighted in previous studies (Xu et al. 2023; Xu et al. 2023; Carr et al. 2016). While ACF is deemed quick, easy to insert, and ideal for learners with a higher success rate (Xu et al. 2023), it is considered necessary for patients requiring rapid fluid resuscitation, highly concentrated intravenous drugs during clinical deterioration, or CT scans with contrast (Thomas et al. 2020). However, ACF insertion is associated with a high failure rate, short dwell time due to occlusion, and accidental removal (Wallis et al. 2014; Carr et al. 2018; Marsh et al. 2021), which warrants consideration. A considerable number of ACF insertions may be attributed to a large cohort of junior ED clinicians and the underutilisation of technology like ultrasound‐guided insertion. Despite efforts to promote awareness and educate staff on its risks, recent Australian research conducted in both ED and inpatient wards suggests that these measures have not effectively addressed the issue (Ruegg et al. 2022). More effective interventions are needed to improve this suboptimal practice.

In our study, we also identified insertion site disinfection as another issue that was previously underreported. ED clinicians often cannulate immediately after cleaning the site with antiseptic wipes, contrary to the recommendation from the Infusion Therapy Standard of Practice (9th version) (Infusion Nurses Society 2024). The Standard advises waiting for the site to air dry completely before insertion, following manufacturers' instructions, because different disinfection products have varying waiting periods. Rushing to apply the dressing without ensuring the site decontaminants have dried can cause additional problems, including skin irritation and poor dressing adherence (Broadhurst et al. 2017). Surprisingly, we observed a high rate of non‐adherence and need for reinforcement of PIVC dressing despite their recent application, which may be due to the site being damp from antiseptic disinfection. Since less than 3% of insertions in our study were emergencies, waiting for complete dryness should be maintained during non‐urgent insertions. Despite being a standard concept in hospital disinfection procedures, research indicates highly variable practices among healthcare workers due to insufficient education and product use instructions (Boyce 2021). Future education initiatives should prioritise emphasising this concept, especially in routine PIVC insertions.

We discovered that ED documentation of the PIVC insertions is frequently incomplete, consistent with global and local studies. An observational study of 40,620 PIVCs from 51 countries revealed that over half lacked documented insertion dates and times (Alexandrou et al. 2018). Similarly, a recent Australian ED study found that only 20% of documentation was fully completed (Thomas et al. 2020). During our chart audit, we were unable to assess if clinicians met Standard 9 (Reviewing ongoing needs) because our electronic medical record system (iEMR) does not include this data. This suggests ongoing need assessment may not be routine in EDs, an important finding given we know many PIVCs sent to the ward are unused, and could potentially have been removed by ED staff. While timely PIVC removal is promoted with tools like I‐DECIDED, mainly in inpatient settings (Ray‐Barruel et al. 2018), its use in ED is limited. Few studies have explored PIVC removal documentation. Although we could not determine how many PIVCs were removed before discharge, our study revealed a very low PIVC removal documentation rate (3.3%), indicating a potential issue with delayed removal or documentation. Premature removal can be problematic if patients need future tests or medications afterwards. Documentation is often seen as an added burden for healthcare workers, especially busy EDs, potentially affecting patient care for procedures like PIVCs (Xu et al. 2024).

### 5.1 Future Research and Practice

To address multiple issues identified in this study, resourced implementation efforts are crucial to promote adherence to evidence‐based practices in dynamic clinical settings. Using strategies and interventions guided by implementation science frameworks can support safe practices and adherence to the national care standard, ensuring quality healthcare. Future research also should explore introducing dedicated vascular access specialist roles (Marsh et al. 2020) in EDs as many clinicians need training, assessment, and support in vascular access device care with a goal to improve patient outcomes and overall quality of care in ED settings.

### 5.2 Strength and Limitations

The strength of this study lies in its large sample size and comprehensive data collection, covering the entire PIVC care process across three EDs with diverse patient cohorts. Observational data was collected at the bedside prospectively by well‐trained ReNs, ensuring accurate measurement and recording. However, the study's limitations include its focus on metropolitan and regional EDs in Australia, potentially limiting generalisability to rural or remote EDs and other settings. Future studies should consider these diverse environments, as clinicians encounter different challenges. We also acknowledge that we may not have accurately captured all PIVC practices due to the limitations of observations conducted during selected shifts and days, which only capture moments of care. Additionally, the self‐selecting nature of the clinician sample, along with the need for formal consent before observations as mandated by the local ethics committee, may introduce both self‐selection sampling bias and the Hawthorne effect among participants (Purssell et al. 2020).

## 6 Conclusion

Our study provides insight into ED PIVC practices and highlights several suboptimal practices requiring attention and improvement, such as patient partnership, insertion practice, and documentation. Addressing these deficiencies in future search and practice is vital to mitigate risks and enhance the quality of patient care.

## Author Contributions

H.(G).X. drafted the research protocol. H.(G).X., A.J.U. and C.M.R. contributed to conceptualisation and design of the study. H.(G).X., L.R. and C.T. contributed to data collection. H.(G).X. and A.D. contributed to data analysis. H.(G).X. drafted the original manuscript. All authors contributed to the critical revision and approval of the final manuscript.

## Ethics Statement

The study has received ethical approval from the Griffith University Human Ethics Committee (2022/025) and Metro South Ethics Committee (HREC/2022/QMS/81524).

## Conflicts of Interest

None to declare. CMR's employer (University of Queensland or Griffith University) has received on her behalf investigator‐initiated research grants from 3 M and Cardinal Health and consultancy funding from 3 M, BBraun and ITL Biomedical, unrelated to this study. AU reports investigator‐initiated research grants paid to her employer from BD, 3 M, Medline and Biolife, unrelated to this study.

## Supporting information


Data S1.

## Data Availability

The data that support the findings of this study are available from the corresponding author upon reasonable request.

## References

[jan16810-bib-0001] Agency for Healthcare Research and Quality . 2013. Guide to Patient and Family Engagement in Hospital Quality and Safety. Agency for Healthcare Research and Quality.

[jan16810-bib-0002] AIHW . 2023. “Time Spent in Emergency Departments, 2012–13 to 2021–22.” https://viz.aihw.gov.au/t/Public/views/2EDAccessBrickUDMTimeinEmergencyDepartments/Table?%3Aembed=y&%3Ahighdpi=false&%3Arender%3Afalse=&%3Atoolbar=n&%3Atabs=n&%3Adisplay_count=n&%3AshowVizHome=n&%3Aorigin=viz_share_link.

[jan16810-bib-0003] Alexandrou, E. , G. Ray‐Barruel , P. J. Carr , et al. 2015. “International Prevalence of the Use of Peripheral Intravenous Catheters.” Journal of Hospital Medicine 10, no. 8: 530–533. 10.1002/jhm.2389. 26041384

[jan16810-bib-0004] Alexandrou, E. , G. Ray‐Barruel , P. J. Carr , et al. 2018. “Use of Short Peripheral Intravenous Catheters: Characteristics, Management, and Outcomes Worldwide.” Journal of Hospital Medicine 13, no. 5: E1–E7. 10.12788/jhm.3039. 29813140

[jan16810-bib-0005] Arts, D. L. , A. G. Voncken , S. Medlock , A. Abu‐Hanna , and H. C. van Weert . 2016. “Reasons for Intentional Guideline Non‐Adherence: A Systematic Review.” International Journal of Medical Informatics 89: 55–62. 10.1016/j.ijmedinf.2016.02.009. 26980359

[jan16810-bib-0006] Australian Commission on Safety and Quality in Health Care . 2021. “Management of Peripheral Intravenous Catheters Clinical Care Standard.” ACSQHC, editor. Sydney.

[jan16810-bib-0007] Australian Commission on Safety and Quality in Health Care . 2022. “Clinical Care Standards: ACSQHC.” https://www.safetyandquality.gov.au/standards/clinical‐care‐standards.

[jan16810-bib-0008] Blonde, L. , K. Khunti , S. B. Harris , C. Meizinger , and N. S. Skolnik . 2018. “Interpretation and Impact of Real‐World Clinical Data for the Practicing Clinician.” Advances in Therapy 35, no. 11: 1763–1774. 10.1007/s12325-018-0805-y. 30357570 PMC6223979

[jan16810-bib-0009] Bombard, Y. , G. R. Baker , E. Orlando , et al. 2018. “Engaging Patients to Improve Quality of Care: A Systematic Review.” Implementation Science 13, no. 1: 98. 10.1186/s13012-018-0784-z. 30045735 PMC6060529

[jan16810-bib-0010] Boyce, J. M. 2021. “A Review of Wipes Used to Disinfect Hard Surfaces in Health Care Facilities.” American Journal of Infection Control 49, no. 1: 104–114. 10.1016/j.ajic.2020.06.183. 32569612

[jan16810-bib-0011] Braithwaite, J. , P. Glasziou , and J. Westbrook . 2020. “The Three Numbers You Need to Know About Healthcare: The 60‐30‐10 Challenge.” BMC Medicine 18, no. 1: 102. 10.1186/s12916-020-01563-4. 32362273 PMC7197142

[jan16810-bib-0012] Broadhurst, D. , N. Moureau , A. J. Ullman , and World Congress of Vascular Access Skin Impairment Management Advisory P . 2017. “Management of Central Venous Access Device‐Associated Skin Impairment: An Evidence‐Based Algorithm.” Journal of Wound, Ostomy, and Continence Nursing 44, no. 3: 211–220. 10.1097/WON.0000000000000322. PMC5417573 28353488

[jan16810-bib-0013] Care Quality Commission . 2014. Our Strategy for Engaging the Public in CQC's Work in 2015–2016. Care Quality Commission.

[jan16810-bib-0014] Carr, P. J. , J. C. Rippey , M. L. Cooke , et al. 2016. “Development of a Clinical Prediction Rule to Improve Peripheral Intravenous Cannulae First Attempt Success in the Emergency Department and Reduce Post Insertion Failure Rates: The Vascular Access Decisions in the Emergency Room (VADER) Study Protocol.” BMJ Open 6, no. 2: e009196. 10.1136/bmjopen-2015-009196. PMC4762116 26868942

[jan16810-bib-0015] Carr, P. J. , J. C. R. Rippey , M. L. Cooke , et al. 2018. “From Insertion to Removal: A Multicenter Survival Analysis of an Admitted Cohort With Peripheral Intravenous Catheters Inserted in the Emergency Department.” Infection Control and Hospital Epidemiology 39, no. 10: 1216–1221. 10.1017/ice.2018.190. 30196798

[jan16810-bib-0016] Clinical Excellence Queensland . 2020. “Promoting Value‐Based Care in EDs (PROV‐ED).” https://clinicalexcellence.qld.gov.au/improvement‐exchange/prov‐ed.

[jan16810-bib-0017] Connell, J. , J. Carlton , A. Grundy , et al. 2018. “The Importance of Content and Face Validity in Instrument Development: Lessons Learnt From Service Users When Developing the Recovering Quality of Life Measure (ReQoL).” Quality of Life Research 27, no. 7: 1893–1902. 10.1007/s11136-018-1847-y. 29675691 PMC5997715

[jan16810-bib-0018] Cooke, M. , A. J. Ullman , G. Ray‐Barruel , M. Wallis , A. Corley , and C. M. Rickard . 2018. “Not “Just” an Intravenous Line: Consumer Perspectives on Peripheral Intravenous Cannulation (PIVC). An International Cross‐Sectional Survey of 25 Countries.” PLoS One 13, no. 2: e0193436. 10.1371/journal.pone.0193436. 29489908 PMC5831386

[jan16810-bib-0019] von Elm, E. , D. G. Altman , M. Egger , et al. 2014. “The Strengthening the Reporting of Observational Studies in Epidemiology (STROBE) Statement: Guidelines for Reporting Observational Studies.” International Journal of Surgery 12, no. 12: 1495–1499. 10.1016/j.ijsu.2014.07.013. 25046131

[jan16810-bib-0020] Harris, P. A. , R. Taylor , B. L. Minor , et al. 2019. “The REDCap Consortium: Building an International Community of Software Platform Partners.” Journal of Biomedical Informatics 95: 103208. 10.1016/j.jbi.2019.103208. 31078660 PMC7254481

[jan16810-bib-0021] Hawkins, T. , J. H. Greenslade , J. Suna , et al. 2018. “Peripheral Intravenous Cannula Insertion and Use in the Emergency Department: An Intervention Study.” Academic Emergency Medicine 25, no. 1: 26–32. 10.1111/acem.13335. 29044739

[jan16810-bib-0022] Infusion Nurses Society . 2024. “Infusion Therapy Standards of Practice.”

[jan16810-bib-0023] Kelly, A.‐M. 2014. “When Is Peripheral Intravenous Catheter Insertion Indicated in the Emergency Department?” Emergency Medicine Australasia 26, no. 5: 51–56. 10.1111/1742-6723.12294. 25186163

[jan16810-bib-0024] Laan, B. J. , J. M. Maaskant , I. J. B. Spijkerman , et al. 2020. “De‐Implementation Strategy to Reduce Inappropriate Use of Intravenous and Urinary Catheters (RICAT): A Multicentre, Prospective, Interrupted Time‐Series and Before and After Study.” Lancet Infectious Diseases 20, no. 7: 864–872. 10.1016/s1473-3099(19)30709-1. 32151333

[jan16810-bib-0025] Lim, Z. J. , D. Nagle , F. McAllan , et al. 2020. “Evaluating the Sustained Effectiveness of a Multimodal Intervention Aimed at Influencing PIVC Insertion Practices in the Emergency Department.” Emergency Medicine Journal 37, no. 7: 444–449. 10.1136/emermed-2019-208852. 32414709

[jan16810-bib-0026] Limm, E. I. , X. Fang , C. Dendle , R. L. Stuart , and D. Egerton Warburton . 2013. “Half of all Peripheral Intravenous Lines in an Australian Tertiary Emergency Department Are Unused: Pain With no Gain?” Annals of Emergency Medicine 62, no. 5: 521–525. 10.1016/j.annemergmed.2013.02.022. 23623052

[jan16810-bib-0027] Marsh, N. , E. Larsen , A. Ullman , et al. 2024. “Peripheral Intravenous Catheter Infection and Failure: A Systematic Review and Meta‐Analysis.” International Journal of Nursing Studies 151: 1–12. 10.1016/j.ijnurstu.2023.104673. 38142634

[jan16810-bib-0028] Marsh, N. , E. Larsen , J. Webster , M. Cooke , and C. Rickard . 2020. “The Benefit of a Vascular Access Specialist Placing a Peripheral Intravenous Catheter: A Narrative Review of the Literature.” Vascular Access 6, no. 1: 15. 10.33235/va.6.1.10-15.

[jan16810-bib-0029] Marsh, N. , E. N. Larsen , M. Takashima , et al. 2021. “Peripheral Intravenous Catheter Failure: A Secondary Analysis of Risks From 11,830 Catheters.” International Journal of Nursing Studies 124: 104095. 10.1016/j.ijnurstu.2021.104095. 34689013

[jan16810-bib-0030] Marzban, S. , M. Najafi , A. Agolli , and E. Ashrafi . 2022. “Impact of Patient Engagement on Healthcare Quality: A Scoping Review.” Journal of Patient Experience 9: 23743735221125439. 10.1177/23743735221125439. 36134145 PMC9483965

[jan16810-bib-0031] Mickan, S. , A. Burls , and P. Glasziou . 2011. “Patterns of ‘leakage’ in the Utilisation of Clinical Guidelines: A Systematic Review.” Postgraduate Medical Journal 87, no. 1032: 670–679. 10.1136/pgmj.2010.116012. 21715571 PMC3181428

[jan16810-bib-0032] Purssell, E. , N. Drey , J. Chudleigh , S. Creedon , and D. J. Gould . 2020. “The Hawthorne Effect on Adherence to Hand Hygiene in Patient Care.” Journal of Hospital Infection 106, no. 2: 311–317. 10.1016/j.jhin.2020.07.028. 32763330

[jan16810-bib-0033] Qualtrics . 2025. “Your Guide to Margin of Error (With Calculator).” https://www.qualtrics.com/en‐au/experience‐management/research/margin‐of‐error/.

[jan16810-bib-0034] R: R Core Team . 2020. R: A Language and Environment for Statistical Computing Vienna. R Foundation for Statistical Computing.

[jan16810-bib-0035] Rangarajan, S. , J. Morgenstern , W. K. Milne , and C. Heitz . 2018. “Hot off the Press: Peripheral Intravenous Cannula Insertion and Use in the Emergency Department.” Academic Emergency Medicine 25, no. 6: 668–671. 10.1111/acem.13390. 29450938

[jan16810-bib-0036] Ray‐Barruel, G. , M. Cooke , M. Mitchell , V. Chopra , and C. M. Rickard . 2018. “Implementing the I‐DECIDED Clinical Decision‐Making Tool for Peripheral Intravenous Catheter Assessment and Safe Removal: Protocol for an Interrupted Time‐Series Study.” BMJ Open 8, no. 6: e021290. 10.1136/bmjopen-2017-021290. PMC5988165 29866733

[jan16810-bib-0037] Reigart, J. R. , K. H. Chamberlain , D. Eldridge , et al. 2012. “Peripheral Intravenous Access in Pediatric Inpatients.” Clinical Pediatrics (Phila) 51, no. 5: 468–472. 10.1177/0009922811435164. 22267855

[jan16810-bib-0038] Rippey, J. C. , P. J. Carr , M. Cooke , N. Higgins , and C. M. Rickard . 2016. “Predicting and Preventing Peripheral Intravenous Cannula Insertion Failure in the Emergency Department: Clinician ‘gestalt’ Wins Again.” Emergency Medicine Australasia 28, no. 6: 658–665. 10.1111/1742-6723.12695. 27862989

[jan16810-bib-0039] Rstudio: RStudio Team . 2019. RStudio: Integrated Development for R. RStudio, Inc.

[jan16810-bib-0040] Ruegg, L. , M. Faucett , A. Clawson , and S. Subedi . 2022. “Reducing the Prevalence of Antecubital Fossa Peripheral Intravenous Cannulation.” British Journal of Nursing 31, no. 2: S8–S14. 10.12968/bjon.2022.31.2.S8 35094536

[jan16810-bib-0041] Thomas, C. , C. J. Cabilan , and A. N. B. Johnston . 2020. “Peripheral Intravenous Cannula Insertion and Use in a Tertiary Hospital Emergency Department: A Cross‐Sectional Study.” Australas Emerg Care 23, no. 3: 166–172. 10.1016/j.auec.2020.02.001. 32139321

[jan16810-bib-0042] Tuffaha, H. W. , C. M. Rickard , J. Webster , et al. 2014. “Cost‐Effectiveness Analysis of Clinically Indicated Versus Routine Replacement of Peripheral Intravenous Catheters.” Applied Health Economics and Health Policy 12, no. 1: 51–58. 10.1007/s40258-013-0077-2. 24408785

[jan16810-bib-0043] Ullman, A. J. , M. Takashima , T. Kleidon , G. Ray‐Barruel , E. Alexandrou , and C. M. Rickard . 2020. “Global Pediatric Peripheral Intravenous Catheter Practice and Performance: A Secondary Analysis of 4206 Catheters.” Journal of Pediatric Nursing 50: 18–25. 10.1016/j.pedn.2019.09.023. 31648879

[jan16810-bib-0044] Wallis, M. C. , M. McGrail , J. Webster , et al. 2014. “Risk Factors for Peripheral Intravenous Catheter Failure: A Multivariate Analysis of Data From a Randomized Controlled Trial.” Infection Control and Hospital Epidemiology 35, no. 1: 63–68. 10.1086/674398. 24334800

[jan16810-bib-0045] Wang, P. S. , J. S. Benner , R. J. Glynn , W. C. Winkelmayer , H. Mogun , and J. Avorn . 2004. “How Well Do Patients Report Noncompliance With Antihypertensive Medications?: A Comparison of Self‐Report Versus Filled Prescriptions.” Pharmacoepidemiology and Drug Safety 13, no. 1: 11–19. 10.1002/pds.819. 14971118

[jan16810-bib-0046] Whalen, M. , B. Maliszewski , and D. L. Baptiste . 2017. “Establishing a Dedicated Difficult Vascular Access Team in the Emergency Department: A Needs Assessment.” Journal of Infusion Nursing 40, no. 3: 149–154. 10.1097/NAN.0000000000000218. 28419011

[jan16810-bib-0047] Xu, H. G. , J. Bowdery , To, Y , et al. 2024. “Peripheral Intravenous Catheter Clinical Care Standard Adherence in Emergency Departments: A Qualitative Study Underpinned by the Behaviour Change Wheel.” Journal of Advanced Nursing 2024: 16409. 10.1111/jan.16409. PMC12535365 39253763

[jan16810-bib-0048] Xu, H. G. , C. Rickard , M. Takashima , M. Butterfield , E. Pink , and A. Ullman . 2023. “Exploring Australian Emergency Department Clinicians' Knowledge, Attitudes, and Adherence to the National Peripheral Intravenous Catheter Clinical Care Standard: A Cross‐Sectional National Survey.” Emergency Medicine Australasia 35, no. 5: 759–770. 10.1111/1742-6723.14214. 37062587

[jan16810-bib-0049] Xu, H. G. , A. Ullman , C. Rickard , and J. Amy . 2023. “Factors Impacting Emergency Department Clinicians' Peripheral Intravenous Catheter Practices; A Qualitative Analysis.” International Emergency Nursing 71: 101366. 10.1016/j.ienj.2023.101366. 37852059

